# Critical Effect of Oxygen Pressure in Pulsed Laser Deposition for Room Temperature and High Performance Amorphous In-Ga-Zn-O Thin Film Transistors

**DOI:** 10.3390/nano12244358

**Published:** 2022-12-07

**Authors:** Yue Zhou, Dao Wang, Yushan Li, Lixin Jing, Shuangjie Li, Xiaodan Chen, Beijing Zhang, Wentao Shuai, Ruiqiang Tao, Xubing Lu, Junming Liu

**Affiliations:** 1 Guangdong Provincial Key Laboratory of Quantum Engineering and Quantum Materials, Institute for Advanced Materials, South China Academy of Advanced Optoelectronics, South China Normal University, Guangzhou 510006, China; 2 College of Science, Qiongtai Normal University, Haikou 571127, China; 3 Laboratory of Solid State Microstructures and Innovation Center of Advanced Microstructures, Nanjing University, Nanjing 210009, China

**Keywords:** a-IGZO, PLD, TFT, oxygen pressure, oxygen vacancy

## Abstract

The aspects of low processing temperature and easy running in oxygen atmosphere contribute to the potential of pulsed laser deposition (PLD) in developing a-IGZO TFTs for flexible applications. However, the realization of low-temperature and high-performance devices with determined strategies requires further exploration. In this work, the effect of oxygen pressure and post-annealing processes and their mechanisms on the performance evolution of a-IGZO TFTs by PLD were systematically studied. A room-temperature a-IGZO TFT with no hysteresis and excellent performances, including a *μ* of 17.19 cm^2^/V·s, an *I*_on_/*I*_off_ of 1.7 × 10^6^, and a SS of 403.23 mV/decade, was prepared at the oxygen pressure of 0.5 Pa. Moreover, an O_2_ annealing atmosphere was confirmed effective for high-quality a-IGZO films deposited at high oxygen pressure (10 Pa), which demonstrates the critical effect of oxygen vacancies, rather than weak bonds, on the device’s performance.

## 1. Introduction

Since Hosono published amorphous In-Ga-Zn-O (a-IGZO) thin film transistors (TFTs) in “Nature” in 2004, considerable attention has been focused on this type of TFT [1]. The In ions in a-IGZO conform to the electronic structure of (*n* − 1)d^10^ns^0^ (*n* ≥ 5). The 5s orbit of In, which has spherical symmetry, overlaps to produce electron channels, so a-IGZO maintains high mobility (≥10 cm^2^/V·s) when it is amorphous [2,3]. Compared with hydrogenated amorphous silicon (a-Si: H), polysilicon, and other ZnO-based oxide semiconductors, a-IGZO has superior electrical characteristics, including high field effect mobility, low sub-threshold swing and high switching current ratio [4,5,6]. Moreover, it can be uniformly deposited over a large area and functionalized on flexible substrates, and thus is promising in applications such as organic light emitting diodes (OLEDs), liquid crystal display (LCD), etc. [7,8,9].

The preparation of a-IGZO thin films in TFTs has been widely demonstrated by magnetron sputtering [10,11] and pulsed laser deposition (PLD) [12,13], while the latter has been acclaimed as particularly suitable for depositing complex oxide films. Although the PLD method has the disadvantages of special target preparation and large-scale film coating, it possesses the advantages of high deposition efficiency, low cost, low processing temperature, easy operation in oxygen atmosphere, and good reproducibility of film chemical composition [1]. Moreover, significant progress in up-scaling has already allowed fabrication of high-quality piezoelectric devices as well as annual fabrication of >100 km of high temperature superconductor (HTS) tape by PLD [14,15,16,17]. Room-temperature a-IGZO TFTs development by PLD has been demonstrated previously for flexible electronics [18,19]; however, the mechanisms of achieving high-performance devices with negligible hysteresis remain elusive or controversial thus far. Moreover, limited knowledge is still available on the effects of oxygen pressure (especially high oxygen pressure [20]), post-annealing temperature, and atmosphere on the deposition of a-IGZO films, even though it is known that they can significantly affect the performance of the devices [21].

In this study, systematic experiments were implemented to explore the performance evolution mechanisms of TFTs with a-IGZO films deposited at different oxygen pressures by PLD. The critical effect of oxygen vacancies, which first decrease and then increase with the increase in oxygen pressure, was confirmed on the mobility (*μ*), threshold voltage (*V*_th_), *I*_on_/*I*_off_, subthreshold slope (SS), and hysteresis window in the transfer curves. Consequently, a room-temperature a-IGZO TFT with no hysteresis and excellent performance, including a *μ* of 17.19 cm^2^/V·s, an *I*_on_/*I*_off_ of 1.7 × 10^6^, and a SS of 403.23 mV/decade, was prepared by PLD at the oxygen pressure of 0.5 Pa, which demonstrates the great potential for the development of a-IGZO TFTs by PLD in future, for instance, in flexible electronics. Moreover, an annealing process in O_2_ atmosphere (350 °C for 1 h) was implemented to improve the performance of devices with a-IGZO films deposited at high oxygen pressure (10 Pa). A *μ* of 9.44 cm^2^/V·s, an *I*_on_/*I*_off_ of 1.6 × 10^8^ and a small hysteresis window of 0.8 V were successfully achieved, which demonstrates the referential significance of our work for achieving optimized a-IGZO properties by simply regulating the oxygen pressure and annealing process during the deposition.

## 2. Experimental

**Figure 1:**
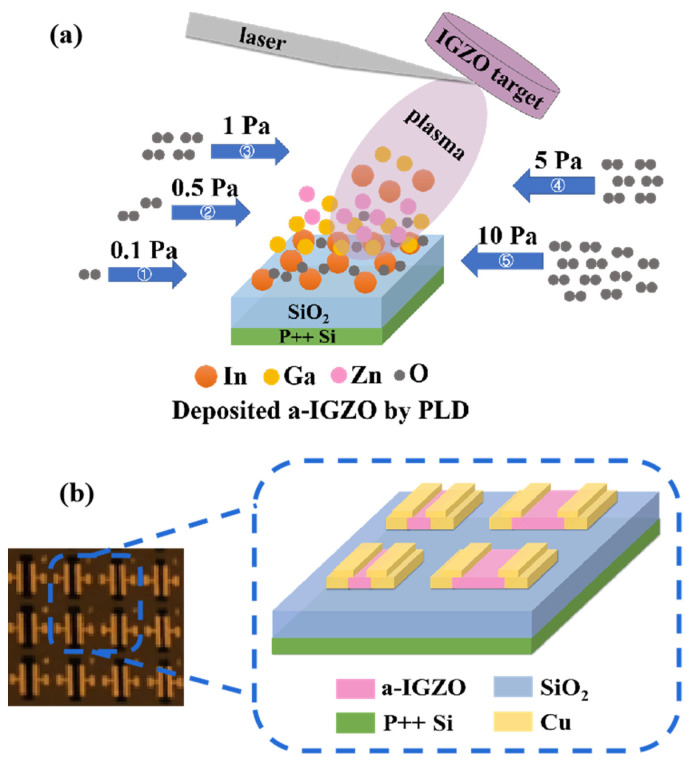
(**a**) PLD preparation of a-IGZO thin films at different oxygen pressures from 0.1 to 10 Pa. (**b**) Schematic and optical image of the device structure of the a-IGZO TFTs.

The a-IGZO TFTs were fabricated on thermally grown SiO_2_ (50 nm)/p^++^-Si substrates, which were cleaned by sonicating in acetone, iso-propyl-alcohol, absolute ethanol, and de-ionized water. The a-IGZO was deposited by pulsed laser deposition (PLD) at room temperature with a thickness of 25 nm. The deposition pulse energy and frequency were 105 mJ and 3 Hz, respectively. The deposition oxygen partial pressure varied from 0.1 to 10 Pa, which is schematically shown in Figure 1a. The channel width was fixed at 1200 μm, and the channel length ranged from 50 μm to 350 μm. The schematic of the device structure and the optical image is exhibited in Figure 1b. After the deposition, the a-IGZO films were annealed at different temperatures (250 to 400 °C) for 1 h. Next, 40-nm Cu films were deposited by a shadow mask to form S/D electrodes.

The surface roughness of the IGZO films was characterized by atomic force microscopy (AFM). To investigate the effects of oxygen pressure on the deposition of the IGZO films, the microstructures were characterized by X-ray diffraction (XRD), and the O-related defects were checked by the X-ray photoelectron spectroscopy (XPS). The electrical characteristics of the fabricated bottom-gate IGZO TFTs were evaluated using a semiconductor parameter analyzer (Agilent, B1500A) at room temperature.

## 3. Results and Discussion

**Figure 2:**
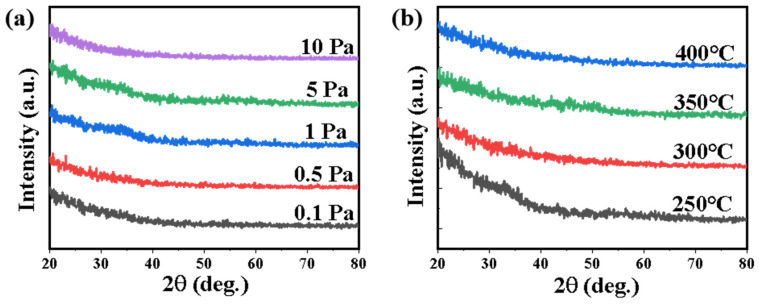
XRD patterns of the IGZO films with different deposition conditions of (**a**) different oxygen pressure varies from 0.1–10 Pa; (**b**) different post-annealing temperature varies from 250–400 °C.

Figure 2a shows the XRD patterns of the 350 °C annealed a-IGZO films deposited on a p^++^-Si wafer at different oxygen pressures (0.1, 0.5, 1, 5 and 10 Pa). No sharp peaks can be identified in the patterns, suggesting a deposition-pressure-independent amorphous feature of the films. The amorphous structure was also confirmed with different post-annealing temperatures (250, 300, 350 and 400 °C) under a fixed oxygen pressure of 1 Pa (Figure 2b), as higher annealing temperature up to ~500 °C was reported previously for the crystallization [19]. Although there is a slight crystallization of IGZO between 20–40 degrees annealed at 250 °C, it does not affect the amorphous property of IGZO in general.

**Figure 3:**
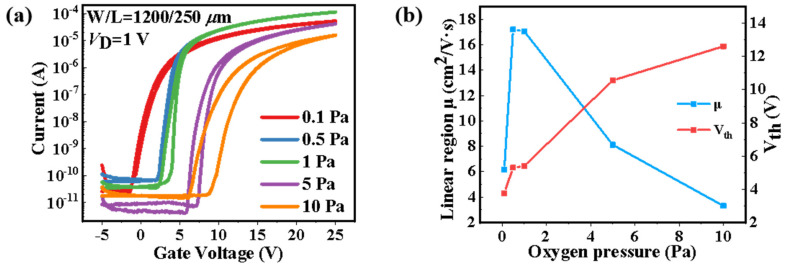
(**a**) Transfer characteristics of the TFTs with a-IGZO thin films deposited at various oxygen pressures. (**b**) Evolution of the device performance (mobility, *V*_th_) extracted from the transfer curves.

Figure 3a illustrates the dual-scan forward and backward transfer characteristics of the a-IGZO TFTs with a *V*_G_ sweeping range from −5 to 25 V (*V*_DS_ = 1 V), where the a-IGZO films were deposited under different oxygen pressure of 0.1, 0.5, 1, 5, and 10 Pa, respectively. To clarify the relationship between the oxygen pressure and the device performance, there was no post-annealing process followed by the film deposition. The key parameters of these TFTs, including linear region mobility (*μ*), sub-threshold swing (SS), on-off current radios (*I*_on_/*I*_off_), and threshold voltage (*V*_th_) are shown in Table 1. The *I*_on_ and *I*_off_ values for all the devices were extracted from the forward sweeping transfer curves, and corresponded to the lowest point of the off state and the highest point of the on state, respectively. The *μ* was extracted from the following equation (for a linear region)

$$\mu =\frac{L}{WC_{i}V_{D}}\frac{\partial I_{D}}{\partial V_{G}}$$

where *L*/*W* is the channel length/width, *C*_i_ is the capacitance per unit area of the gate insulator (50 nm SiO_2_), and *I*_D_, *V*_D_, and *V*_G_ are the drain current, drain voltage and gate voltage, respectively.

It can be seen from Figure 3b that the *μ* first increased and then decreased, while the *V*_th_ increased markedly with the increase in oxygen pressure. The positive shift of the *V*_th_ might be ascribed to the decreasing of electron carrier concentration, which seemed to be accord with the filling of the oxygen vacancies with increased oxygen pressure. It has been reported previously that the decrease in oxygen vacancy will contribute the decrease in carrier concentration and the increase in carrier mobility in a-IGZO channel layer [22,23]. However, the evolution of the *μ* is unlikely to be dominated by the concentration of oxygen vacancies, as a simultaneous decrease in carrier mobility and concentration has been observed in devices with oxygen pressures from 0.5 to 10 Pa. Moreover, the oxygen vacancies may not simply decrease with the increase in oxygen pressure, which is evidenced by the evolution of the clockwise hysteresis windows in the dual-scan transfer curves. The windows enlarge with the increase in oxygen pressures from 0.5 to 10 Pa, which indicates pronounced charge trapping induced in a device with high oxygen pressure. Note that a room-temperature-prepared device with no hysteresis and excellent performance, including a *μ* of 17.19 cm^2^/V·s, an *I*_on_/*I*_off_ of 1.7 × 10^6^, and a SS of 403.23 mV/decade, was obtained at the oxygen pressure of 0.5 Pa, which demonstrates great potential for the development of a-IGZO TFTs by PLD in the future, for instance, in flexible electronics.

**Figure 4:**
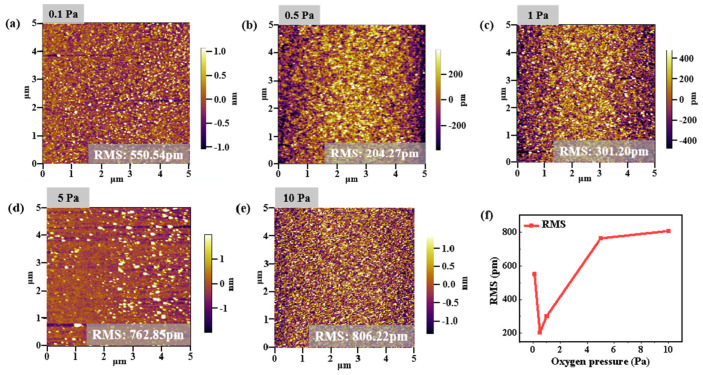
AFM morphology images of the a-IGZO films deposited at different oxygen pressures of (**a**) 0.1 Pa, (**b**) 0.5 Pa, (**c**) 1 Pa, (**d**) 5 Pa, and (**e**) 10 Pa, respectively. (**f**) Evolution of the RMS extracted from the AFM morphology images.

The AFM images of the films grown at different oxygen pressures (0.1 Pa, 0.5 Pa, 1 Pa, 5 Pa, and 10 Pa) are shown in Figure 4, in which the root mean square (RMS) roughness values are 550.54 pm, 204.27 pm, 301.20 pm, 762.85 pm, and 806.22 pm, respectively. The general evolution tendency of the roughness and the mobility were confirmed as consistent. The smoothest surface can be observed with an oxygen pressure of 0.5 Pa, which indicates the high quality of the a-IGZO film, and has been demonstrated to have an optimized device performance. As mentioned above, all the IGZO films we deposited were amorphous. The roughness in crystalline films can be affected by the crystallinity, grain size, etc. [24,25]. The roughness of amorphous films tends to be highly related to the deposition pressure. The collision with more oxygen molecules decreases the kinetic energy of the ablated species, thus leading to the aggregation of the adatoms or clusters and increasing the roughness by reducing the migration ability of the adatoms [26]. The decrease in roughness with oxygen pressures between 0.1 and 0.5 Pa might result from the filling of oxygen vacancies at high oxygen pressure. On the other hand, PLD is a strongly nonequilibrium process. In nonequilibrium surface growth processes, kinetic roughening plays a significant role in determining surface roughness. It is not the aggregation of adatoms, but the decrease in the mean path of adsorbed specie movement at the surface that decreases film roughness. Then a film nucleates by islands, which then grow in size till reaching coalescence, and the surface roughness diminishes, as in Figure 4f.

**Figure 5:**
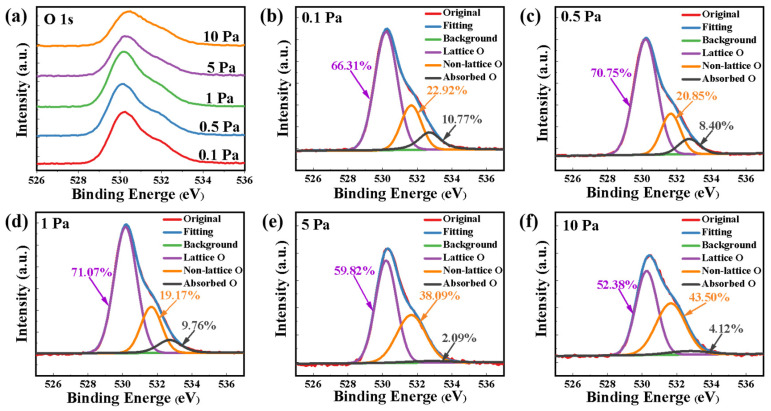
(**a**) The O 1s XPS spectra for a-IGZO films deposited at different oxygen pressures. Gaussian fitting of O 1s spectra for a-IGZO films deposited at pressures of (**b**) 0.1 Pa, (**c**) 0.5 Pa, (**d**) 1 Pa, (**e**) 5 Pa, and (**f**) 10 Pa, respectively.

The mechanisms are further evidenced in the X-ray photoelectron spectroscopy (XPS) measurements. Figure 5a shows the results of the O 1s XPS spectra of the a-IGZO films fabricated at different oxygen pressures. The spectra are carefully decomposed into three peak shapes using Gaussian fits, which reflect different types of O species in the samples as shown in Figure 5b–f [27,28]. The low binding energy component located at 530.2 eV is usually attributed to O^2−^ ions surrounded by Zn, Ga, and In atoms in the IGZO compound system (lattice O). The peak at the intermediate binding energy around 531.65 eV is attributed to oxygen-deficient regions of the IGZO matrix (non-lattice O). It represents the oxygen atom close to the oxygen vacancy, interstitial oxygen, or other related defects. The higher binding energy peak around 532.7 eV correlates to the absorbed oxygen on the surface (adsorbed O) such as −CO_3_ or adsorbed O_2_, which is related to the exposure time of the sample in the air. The fraction of non-lattice O first decreased and then increased with the increase in the oxygen pressure, which generally corresponded to the evolution of the mobility and the hysteresis window in the transfer curves shown in Figure 3a. The fraction of non-lattice O also clearly correlates with surface roughness. With the ratio of non-lattice O decreasing from 22.92 to 20.85% shown in Figure 5b,c, the surface roughness also decreased from 550.54 to 204.27 pm. When both the non-lattice O and the film roughness are minimal, the a-IGZO film has better properties and better device performance. A larger surface roughness provides more sites for weak oxygen adsorption. Non-lattice O increases with the increase in a-IGZO film roughness, resulting in poor device performance.

**Figure 6:**
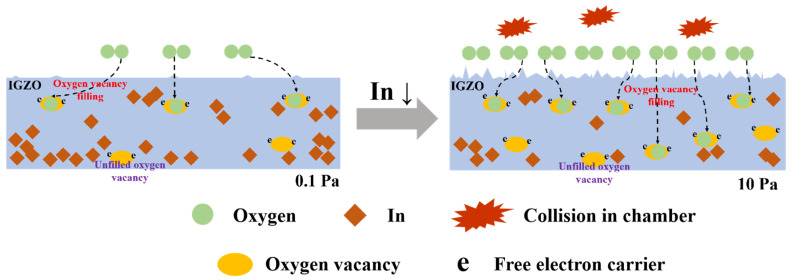
Schematic showing mechanism of a-IGZO films deposited by PLD with different oxygen pressures.

With the oxygen pressure between 0.5 to 10 Pa, the oxygen vacancy generally increases, which does not conform to the positive shift of the *V*_th_ in Figure 3a, as the formation of oxygen vacancy will release electrons, which increases the carrier concentration of the channel. This contradiction can be explained by the change in chemical composition reported in a-IGZO films deposited by PLD, in which the proportion of In atoms is gradually decreased with the increase in oxygen pressure [29,30]. The overlap of the 5s orbits of In produces electron channels in a-IGZO, thus the decreased In ratio will lead to the decrease in both carrier mobility and concentration, which fits well with the results in our work. On the whole, we believe that the effect of oxygen pressure on both the oxygen vacancy and the chemical composition in a-IGZO films determines the electrical performance, and the mechanisms are schematically shown in Figure 6.

**Figure 7:**
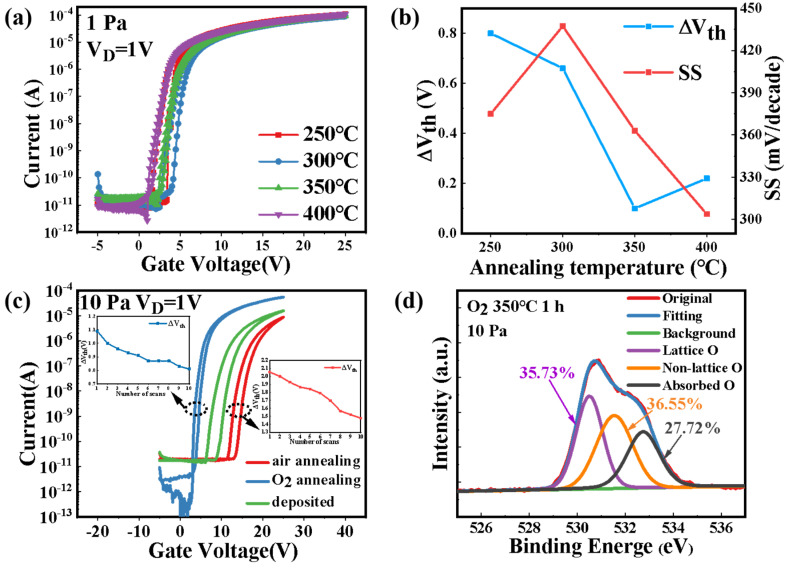
(**a**) The transfer characteristics of the a-IGZO-TFTs with various annealing temperature. (**b**) Evolution of the performances (Δ*V*_th_, SS) extracted from the transfer curves. (**c**) The transfer characteristics of TFTs with a-IGZO films deposited at a high oxygen pressure of 10 Pa, followed by annealing in different atmospheres. (**d**) The O 1s XPS spectra for a-IGZO films deposited at 10 Pa and annealing in O_2_ for 1 h.

We also discussed the effect of annealing processes on the electrical performance. The TFTs with a-IGZO films grown at 1 Pa oxygen pressure were annealed in air at 250 °C, 300 °C, 350 °C, 400 °C, respectively. Figure 7a shows the dual-scan transfer characteristics of the with a *V*_G_ sweeping range from −5 to 25 V (*V*_DS_ = 1 V). The parameter evolution of the hysteresis window size and the SS is shown in Figure 7b. The devices with annealing temperature over 350 °C were observed to have a small hysteresis window (∆*V*_th_ = 0.1 V) and SS (330 mV/decade), indicating the repairing of some oxygen defects, including oxygen vacancy and weakly bonded O atoms at the channel/dielectric interface during the annealing processes [30].

The transfer characteristics of the devices with a-IGZO deposited at a high oxygen pressure of 10 Pa followed by annealing in different atmosphere, are shown in Figure 7c. The device annealed in oxygen for 1 h had significantly improved mobility from 3.31 to 9.44 cm^2^/*V*·s, and *I*_on_/*I*_off_ from 1.1 × 10^6^ to 1.6 × 10^8^. Note that reduced ∆*V*_th_ of 0.8 V after 10 sweeping cycles can be observed, while the device annealed in air for 1 h had a ∆*V*_th_ of 1.5 V. The advanced performance of the device annealed in O_2_ atmosphere indicates the critical effect of oxygen vacancy on the electrical properties of a-IGZO thin films deposited at high oxygen pressure. The effect of oxygen annealing on the performance of devices depends on the defects existed in the semiconductors. The presence of excess O atoms was demonstrated previously in a-IGZO thin films deposited at high oxygen pressures by PLD. The contents of both weakly bonded O atoms and oxygen vacancies increase with the deposition pressure. However, the content of weakly bonded O atoms might be increased in O_2_ atmosphere, as it can be reformed by the migration of O_2_ into the films. The repairing of the oxygen vacancies can be evidenced by the XPS measurement shown in Figure 7d, in which a decreased non-lattice oxygen ratio (36.55%) can be found in the O 1s spectra for the a-IGZO film deposited at 10 Pa and annealed in O_2_ for 1 h. This observation is referential for achieving optimized a-IGZO properties by simply regulating oxygen pressure and annealing process during the deposition.

## 4. Conclusions

In this work, a room-temperature a-IGZO TFT with no hysteresis and excellent performance, including a *μ* of 17.19 cm^2^/V·s, an *I*_on_/*I*_off_ of 1.7 × 10^6^, and an SS of 403.23 mV/decade, was prepared by PLD at the oxygen pressure of 0.5 Pa, which demonstrates the great potential for developing a-IGZO TFTs by PLD in future, for instance, in flexible electronics. The evolution mechanisms of the key performances in developed TFTs with a-IGZO films deposited by PLD at different oxygen pressures were clarified. The *μ*, *V*_th_, *I*_on_/*I*_off_, SS, hysteresis window and even the surface roughness are all highly related to the oxygen pressure. The critical effect of oxygen vacancies on device performance, and the filling and reforming of oxygen vacancies at high oxygen pressures were discussed in detail. According to the proposed mechanism, an annealing process in O_2_ atmosphere (350 °C for 1 h) was implemented to improve the performance of devices with a-IGZO films deposited at high oxygen pressure (10 Pa). A mobility of 9.44 cm^2^/V·s, an *I*_on_/*I*_off_ of 1.6 × 10^8^ and a small hysteresis window of 0.8 V were successfully achieved, demonstrating the referential significance of our work for achieving optimized a-IGZO properties by simply regulating oxygen pressure and annealing process during the deposition.

## Figures and Tables

**Table 1:** Summary of the performances of *μ*, *V*_th_, SS, and *I*_on_/*I*_off_, in TFTs with a-IGZO thin films deposited at different oxygen pressures.

Oxygen Pressure (Pa)	*μ* (cm^2^/V·s)	*V*_th_ (V)	SS (mV/Decade)	*I*_on_/*I*_off_
0.1	6.15	3.77	580.38	1.2 × 10^6^
0.5	17.19	5.3	403.23	1.7 × 10^6^
1	17.07	5.4	469.39	3.0 × 10^6^
5	8.12	10.6	487.80	1.0 × 10^7^
10	3.31	12.6	685.73	1.1 × 10^6^

## Data Availability

Data presented in this article will be available upon request.
